# Regenerative Medicine: Taming the Chimaera

**DOI:** 10.1016/j.stemcr.2018.09.003

**Published:** 2018-10-09

**Authors:** Bernard A.J. Roelen

**Affiliations:** 1 Department of Farm Animal Health, Faculty of Veterinary Medicine, Utrecht University, Yalelaan 104, 3584CM Utrecht, the Netherlands

## Abstract

In this issue of *Stem Cell Reports*, Hamanaka et al. (2018) describe the generation of chimeric mice with all vascular endothelial cells derived from pluripotent stem cells. This approach is desirable to prevent immune rejection when human stem cells are combined with animal embryos to grow human organs in animals.

## Main Text

One of the main goals of the stem cell and regenerative medicine fields is the generation of functional tissues or organs to be used for transplantation to the human body. Although the information needed to form all tissue types and how these need to interact is present in the genomes of almost all cells, the differentiation and *in vitro* culture of complex three-dimensional structures with different tissue types, such as organs, has proven to be rather difficult indeed (Peloso et al., 2015). One way to approach this formidable task is to carefully observe nature and in particular embryology: identifying the signaling pathways, processes, and structures active in developing embryos during organ formation. This knowledge can subsequently be used to mimic the processes *in vitro* from stem cells (Gilmour et al., 2017). The activation of the various pathways and interactions between the cells is of such an overwhelming complexity that it seems an arduous task to replicate this *in vitro*. On the other hand, in developing embryos these processes are carried out seemingly effortlessly.

When two embryos at an early stage of development are combined they can develop to form one single organism. The animal formed is called a chimera, in reference to the fearsome fire-breathing creature of Homer’s Iliad with the head of a lion, a goat’s head growing out of the back, and a serpent as a tail. In modern-day biology, a chimera is defined as an animal that contains two or more genetically distinct cell types that is derived from more than one zygote. In this respect chimerism should not be confused with mosaicism, which is defined by a combination of cells with different genotypes originating from one single oocyte (McLaren, 1974). Chimeras can form spontaneously, also in humans, when two fertilized eggs, instead of developing separately as fraternal twins, amalgamate. Naturally occurring chimeras are completely viable and fertile, except for XX/XY chimeras, which are usually infertile.

In stem cell biology, chimera formation can be particularly rewarding when pluripotent stem cells (PSCs) are combined with an embryo to define the level of pluripotency of the stem cells and to create gene knockout animals. When embryonic cells and pluripotent cells are combined, the contribution to the different tissue types is usually random. The system can be directed in such a way, however, as to produce whole tissue types or organs entirely derived from stem cells. In a procedure called blastocyst complementation, embryos that are genetically deficient to form a cell type or organ are combined with stem cells that can form the targeted organ. Even more radically, when embryo and PSCs are from different species, an interspecific chimera can be formed, and since mammalian PSCs will not contribute to the extra-embryonic lineages, uterine transplantation can lead to living interspecific chimeric animals. By combining mouse embryos genetically deficient for *Pdx1*, and therefore unable to form a pancreas, with rat PSCs, mouse-rat chimeras were born with a normally functioning rat pancreas (Kobayashi et al., 2010). Theoretically this offers unique opportunities for the use of stem cells in regenerative medicine and indeed it has been proposed to produce functional human organs from PSCs in a developing embryo of another species, for instance a livestock species (Suchy and Nakauchi, 2017, Wu et al., 2017). When patient-specific PSCs are used, like induced pluripotent stem cells (iPSCs), could the PSC-derived organ be used for transplantation without eliciting an immune response? Not quite, since although in the case of the mouse with the rat pancreas all pancreatic cells were of rat origin, the non-pancreatic lineages such as the blood vessels were a mixture or mouse and rat cells. To avoid an immune response it is critical to generate organs in which the vascular endothelial cells are derived from the PSCs. In this issue of *Stem Cell Reports*, Hamanaka and colleagues explored the possibilities of generating mice in which the blood vessels contained entirely PSC-derived vascular endothelial cells (Hamanaka et al., 2018).

**Figure 1:**
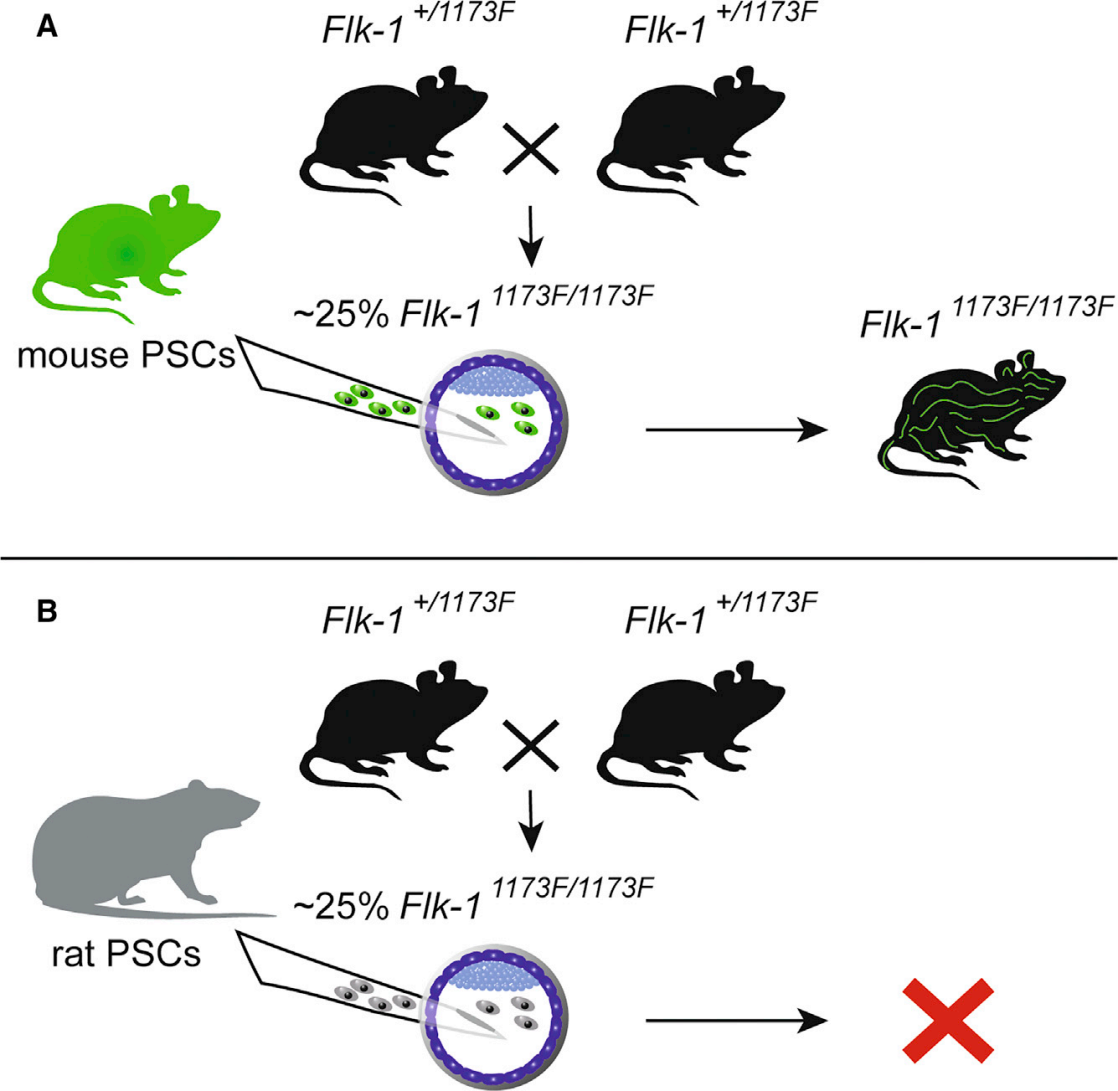
Wild-Type Cells Can Form Vascular Endothelial Cells in Mice with a Disrupted Vascular Endothelial Growth Factor Receptor 2 (A) Intercrosses of *Flk-1*^*+/1173F*^ mice result in approximately 25% of *Flk-1*^*1173F/1173F*^ blastocysts. Injection of these blastocysts with mouse pluripotent stem cells (PSCs) gives rise to viable chimeric mice with all vascular endothelial cells derived from the PSCs. (B) Injection of *Flk-1*^*1173F/1173F*^ mouse blastocysts with rat PSCs did not result in chimeric animals born. All embryos died in utero before embryonic day 13.5.

First, embryos had to be formed without proper vasculogenesis. For this Hamanaka and colleagues made use of mice with an abnormal vascular endothelial growth factor (VEGF) receptor 2. This single transmembrane receptor can bind four members of the VEGF family and regulates vascular endothelial cell function after autophosphorylation upon VEGF binding. *Flk-1* is the murine gene coding for the receptor and *Flk-1*-deficient embryos die in utero around embryonic day (E) 8.5 due to the absence of endothelial and hematopoietic cells. Mice in which tryrosine 1173 of VEGF receptor 2 has been genetically replaced by phenylalanine, *Flk-1*^*1173F/1173F*^ mice, have the same phenotype as the gene knockout animals (Sakurai et al., 2005). Similarly, green fluorescent protein (GFP)-labeled *Flk-1*^*1173F/1173F*^ iPSCs were not able to contribute to blood vessels in chimeric animals formed with wild-type embryos, as demonstrated by absence of PECAM1 immunofluorescence in the GFP-labeled cells. Importantly, the *Flk-1*^*1173F/1173F*^ iPSCs did contribute to other cells and organs in the mouse chimera, demonstrating the pluripotency of the cells. In a reversed experiment, blastocysts from *Flk-1*^*+/1173F*^ intercrosses were injected with GFP-labeled iPSCs, which resulted in living *Flk-1*^*1173F/*1173F^ mice with the vascular endothelial cells derived from the introduced cells (Figure 1A). Other tissues were found to be a mixture of host and introduced cells. FACS analysis demonstrated that the entire vascular endothelial population, identified by absence of CD45 and expression of PECAM1, was iPSC derived. Disappointingly, although it has been firmly established that healthy mouse-rat interspecific chimeras can be formed, no live animals were born after injection of rat iPSCs into *Flk-1*^*1173F/117*3F^ mouse blastocysts (Figure 1B). Embryos were recovered at E9.5, slightly older than mouse *Flk-1*^*1137F/1137F*^ embryos can reach, but at E13.5 apparently all chimeras had died in utero. It would be important to determine whether rat PSCs indeed formed vascular endothelial cells in the mouse-rat chimera and if so why the chimeric embryos terminated development.

Interestingly not only the vascular endothelial cells but also all hematopoietic stem cells in the bone marrow of the *Flk-1*^*1173F/1173F*^ mouse chimera were stem cell derived, as determined by GFP labeling. To next test the ability of these cells to reconstitute the bone marrow, the authors transplanted the bone marrow of *Flk-1*^*1173F/1173F*^ chimeras into irradiated mice, where the cells differentiated into three hematopoietic lineages. This is a crucial requirement of organs made in interspecies chimeras to be used for transplantation; to reduce the risk of immune-rejection, it would be important that the organ in question, as well as the blood vessels and residual hematopoietic cells, is recognized by the patient’s immune system as belonging to the self.

The feasibility of generating all vascular endothelial cells and hematopoietic cells in chimeric animals from PSCs by blastocyst complementation is an important step forward for the generation of human transplantable organs in animals. At the same time, it demonstrates the complexity of this procedure. First the formation of a particular organ or tissue should be genetically prevented in the host blastocyst. Blood vessel and blood formation should also be inhibited so that these are formed by the patient-specific PSCs. The results obtained by Hamanaka and colleagues first demonstrate the complexity even in intraspecies chimeras (Hamanaka et al., 2018). Since mice without vascular endothelial cells are not viable, *Flk-1*^*1173F/1173F*^ blastocysts could only be derived by intercrosses at a success rate of around 25%. Strikingly, the percentage of viable embryos obtained after blastocyst complementation using mouse PSCs was lower, indicating that PSCs do not always fill the available niche. This should be further investigated so that we can understand whether the number of vascular endothelial cells was simply too low to make sufficient blood vessels and whether the number of vascular endothelial cells is related to the overall percentage of chimerism.

A next key goal would be the generation of an organ, for instance pancreas, and vascular endothelial and hematopoietic cells using blastocyst complementation with mouse iPSCs, and the transplantation of that organ into another mouse. Equally important would be unraveling why rat PSCs could not rescue the *Flk-1*^*1173F/1173F*^ mice. Possibly a critical vascular endothelial cell number is needed for survival, or major differences exist between ligand-receptor interactions of the two species.

The paper by Hamanaka and colleagues is important for the generation of organs in animals, but at the same time it shows us that there are many questions that need to be addressed before this can become reality. It also makes it evident that a significant amount of genetic creativity is demanded to ensure that the right cell types are formed at the correct positions. For the 21^st^ century Bellerophon, the aim is not to kill the Chimaera but to exactly control which structures have the needed specifications. The fact that vascular endothelial cells were not formed in interspecific chimeras of the evolutionarily closely related mouse and rat indicates the challenge of accomplishing the growth of human organs in more distantly related species such as pigs.

## References

[bib1] Gilmour D., Rembold M., Leptin M. (2017). From morphogen to morphogenesis and back. Nature.

[bib2] Hamanaka S., Umino A., Sato H., Hayama T., Yanagida A., Mizuno N., Kobayashi T., Kasai M., Suchy F.P., Yamazaki S. (2018). Generation of vascular endothelial cells and hematopoietic cells by blastocyst complementation. Stem Cell Reports.

[bib3] Kobayashi T., Yamaguchi T., Hamanaka S., Kato-Itoh M., Yamazaki Y., Ibata M., Sato H., Lee Y.S., Usui J., Knisely A.S. (2010). Generation of rat pancreas in mouse by interspecific blastocyst injection of pluripotent stem cells. Cell.

[bib4] McLaren A. (1974). Mammalian Chimaeras.

[bib5] Peloso A., Dhal A., Zambon J.P., Li P., Orlando G., Atala A., Soker S. (2015). Current achievements and future perspectives in whole-organ bioengineering. Stem Cell Res. Ther..

[bib6] Sakurai Y., Ohgimoto K., Kataoka Y., Yoshida N., Shibuya M. (2005). Essential role of Flk-1 (VEGF receptor 2) tyrosine residue 1173 in vasculogenesis in mice. Proc. Natl. Acad. Sci. USA.

[bib7] Suchy F., Nakauchi H. (2017). Lessons from interspecies chimeras. Annu. Rev. Cell Dev. Biol..

[bib8] Wu J., Platero-Luengo A., Sakurai M., Sugawara A., Gil M.A., Yamauchi T., Suzuki K., Bogliotti Y.S., Cuello C., Morales Valencia M. (2017). Interspecies chimerism with mammalian pluripotent stem cells. Cell.

